# Three‐stage approach for aortoesophageal fistula after Roux‐en‐Y esophagojejunostomy

**DOI:** 10.1111/1759-7714.14446

**Published:** 2022-04-25

**Authors:** Shuo‐Ying Dai, Chun‐Hao Chang, Yi‐Chen Wang, Jih‐Sheng Wen, Ting‐Kai Liao, Wei‐Ting Lin, Ren‐Hao Chan, Meng‐Ta Tsai, Wei‐Li Huang

**Affiliations:** ^1^ Division of Thoracic Surgery, Department of Surgery National Cheng Kung University Hospital, College of Medicine, National Cheng Kung University Tainan Taiwan; ^2^ Division of Cardiovascular Surgery, Department of Surgery National Cheng Kung University Hospital, College of Medicine, National Cheng Kung University Tainan Taiwan; ^3^ Division of General Surgery, Department of Surgery National Cheng Kung University Hospital, College of Medicine, National Cheng Kung University Tainan Taiwan; ^4^ Division of Colorectal Surgery, Department of Surgery National Cheng Kung University Hospital, College of Medicine, National Cheng Kung University Tainan Taiwan

**Keywords:** aortoesophageal fistula, esophagojejunostomy, three‐stage approach

## Abstract

Surgical management of post‐esophagojejunostomy aortoesophageal fistula (AEF) has been scarcely reported, but is universally fatal. This report described a case of AEF after total gastrectomy with Roux‐en‐Y esophagojejunostomy and adjuvant chemoradiotherapy for gastric cardiac cancer. A three‐stage hybrid approach was used to successfully manage this complication. First, thoracic endovascular aortic repair curbed bleeding. Second, radical fistula resection eradicated infected areas and adjacent structures. Third, esophageal reconstruction using an ileocolonic conduit restored gastrointestinal continuity. This strategy could be safely feasible for managing post‐esophagojejunostomy AEF.

## Graphical abstract

This report described a case of aortoesophageal fistula (AEF) after total gastrectomy with Roux‐en‐Y esophagojejunostomy and adjuvant chemoradiotherapy for gastric cardiac cancer. A three‐stage hybrid‐approach was used to successfully manage this complication, as follows: (1) bridging thoracic endovascular aortic repair controls bleeding and stabilizes hemodynamics; (2) radical fistula resection eradicates infectious source and fragile structures; (3) esophageal reconstruction using an ileo‐colon conduit restores gastrointestinal continuity. This strategy could be safe and feasible for managing post‐esophagojejunostomy AEF.

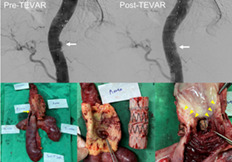

## INTRODUCTION

Aortoesophageal fistula (AEF) is rare, but life‐threatening. The management of secondary AEF is even more challenging because severe adhesions caused by previous operation. The optimal strategy remains controversial. We report a case of post‐esophagojejunostomy AEF, which was successfully managed via a three‐stage approach.

## CASE REPORT

In January 2019, a 67‐year‐old man with gastric cardiac cancer underwent total gastrectomy and Roux‐en‐Y esophagojejunostomy via a circular staple technique in a hospital. The pathological result showed signet‐ring cell carcinoma, pT3N3aM0, stage IIIB, with positive proximal margins; hence, adjuvant chemoradiotherapy was performed.

**FIGURE 1:**
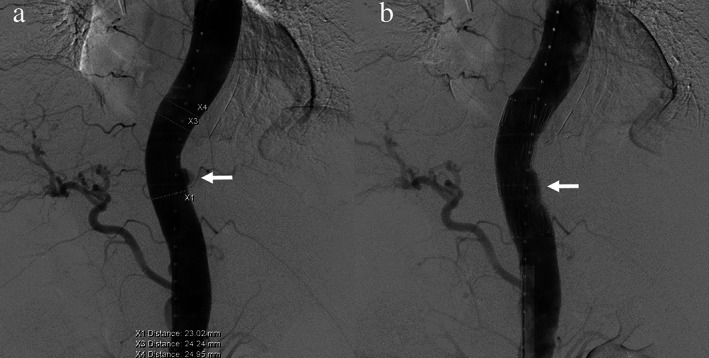
A 67‐year‐old male with gastric cardiac cancer suffered from massive hematemesis and hemorrhagic shock after total gastrectomy and esophagojejunostomy. Urgent aortic angiography showed an aortoesophageal fistula with pseudoaneurysm (white arrow) and active bleeding into the bowel lumen, which was controlled via thoracic endovascular aortic repair (TEVAR) with a stent graft (cook zenith 30 mm diameter × 8.2 cm). (a) Pre‐TEVAR. (b) Post‐TEVAR.

After 4 months of surveillance, he was admitted to the emergency department of our hospital for intermittent bloody stool; panendoscopy showed ulceration near anastomosis without evidence of local recurrence. Massive hematemesis with hemorrhagic shock occurred 9 months after primary surgery, and panendoscopy revealed one marginal ulcer with spurting bleeding over the esophagojejunal anastomosis. Urgent aortic angiography confirmed the AEF. Thoracic endovascular aortic repair (TEVAR) with a stent graft helped control bleeding (Figure 1).

**FIGURE 2:**
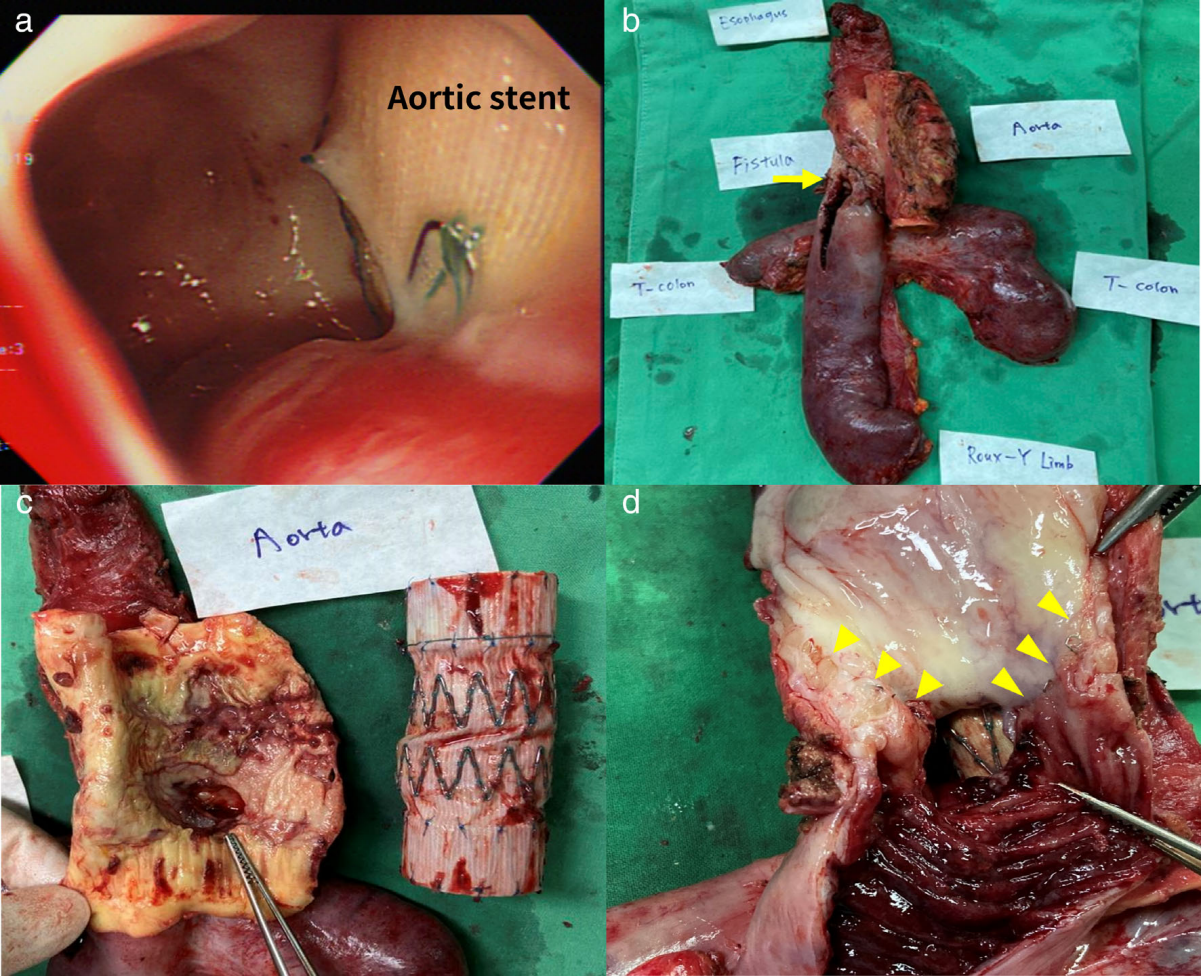
Panendoscopy performed 23 days after aorta stenting showed aortoesophageal fistula deterioration with aortic stent exposure (a). The patient underwent en bloc resection of the fistula (yellow arrow) and surrounding structures (b). The fistula had a gross diameter of 1 cm (c). The fistula was located at the junction of the esophagojejunostomy with visible surgical staples (yellow arrowheads) (d).

After aortic stenting, the patient's hemodynamic status was stable and there was no bleeding. He was kept at nil per os under parenteral nutrition support for ~2 weeks with the hope that the esophageal defect would heal spontaneously. However, a spiking fever was noticed after resuming oral intake; moreover, panendoscopy revealed a deteriorated AEF with an exposed aortic stent (Figure 2(a)). After multidisciplinary team consultation, radical resection of the AEF was intended to control infection and prevent further bleeding. First, the thoracic esophagus was dissected from adjacent structures via right video‐assisted thoracoscopic surgery. Second, the Roux limb and segmental transverse colon near the spleen flexure were resected simultaneously because severe and unbreakable adhesions via laparotomy. Finally, after a left thoracoabdominal incision with left heart bypass and cross‐clamping at the mid‐thoracic aorta and above the celiac trunk, en‐bloc resection of the AEF with aorta and stent, esophagus, Roux limb, and transverse colon was completed (Figure 2(b)). Next, descending thoracic aorta reconstruction with a 25‐mm graft was done.

On gross examination, the AEF had a 1‐cm defect in diameter and presented at the esophagojejunal anastomosis line (Figure 2(c)). The pathology verified fistula formation and acute suppurative inflammation without evidence of malignancy (Figure 2(d)). The patient completed antibiotic therapy and was discharged after a 59‐day hospital stay.

**FIGURE 3:**
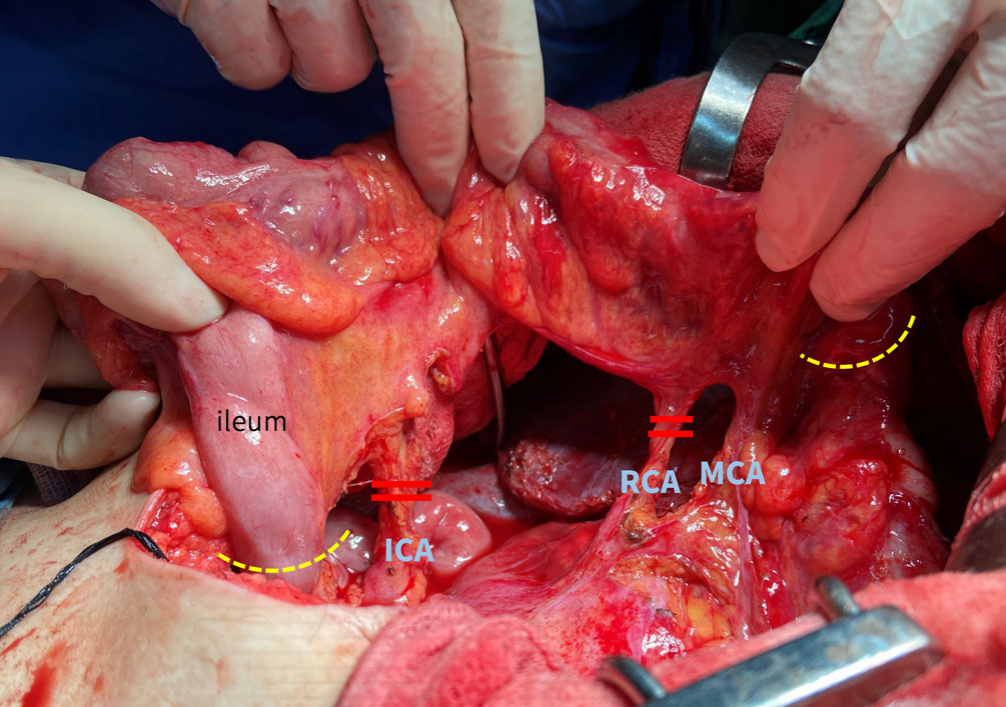
A neoesophagus was constructed via an ileocolonic conduit pedicled on the middle colic artery. ICA, ileocolic artery; RCA, right colic artery; MCA, middle colic artery (right branch).

At the 1‐year follow‐up, the patient underwent esophageal reconstruction using an ileo‐colon conduit (Figure 3). First, colorectal surgeons released the dense adhesions around the transverse colon to facilitate conduit harvesting and further ileocolonic anastomosis. A neoesophagus was constructed using an ileo‐colon graft pedicled on the middle colic artery and pulled up to the left cervical region via the retrosternal route for esophagus ileostomy. He was discharged after a 2‐week hospitalization. He now lives independently and is regularly followed up without tumor recurrence.

## DISCUSSION

AEF is rare, but life‐threatening and often diagnosed by autopsy.^1^ Thoracic aortic aneurysms constitute most cases of primary AEFs. In contrast, secondary AEFs are uncommon challenging complications usually from prior operations, like TEVAR or esophageal surgery.^2^ Secondary AEFs comprise about 5% of all AEFs, with an incidence of 1.9% after esophagogastrectomy.^1^ Post‐esophagogastrectomy AEFs were strongly related to enteric anastomosis leakage, with most (82%) occurring 2 to 6 weeks postoperatively.^1^

Only a few post‐esophagojejunostomy AEFs have been reported. Molina‐Navaro et al.^1^ analyzed 23 cases of aortogastric fistula after esophagogastrectomy for esophageal cancer and found only one (4.3%) patient survived this lethal complication. Sato et al.^3^ also reported a patient with aortogastric fistula 46 days after esophagectomy and intrathoracic esophagogastric anastomosis. The fistula was successfully obliterated via primary closure twice and sequential TEVAR.^3^ By contrast, only two patients with post‐esophagojejunostomy aortoesophageal fistula were reported.^4^, ^5^ The survivor, reported by Okita et al.,^4^ underwent primary closure and TEVAR to manage AEF, which occurred 24 days after esophagogastrectomy and alimentary reconstruction using a free jejunal graft. The other case, reported by Honda et al.,^5^ died 11 days after esophagogastrectomy, and the diagnosis of AEF was confirmed on autopsy. In our case, there was a 9‐month interval between primary surgery and the development of AEF, and the patient also underwent adjuvant radiotherapy, which would deteriorate fistula formation and hinder subsequent surgical procedures. This is the first report on concomitant transverse colon resection with radical AEF resection; moreover, we demonstrated the feasibility of using an ileocolonic conduit even after segmental transverse colectomy. Two years after esophagogastrectomy for cardiac cancer, the patient was alive and without recurrence.

Conservative therapies cause most patients with AEF to die of infectious complications or recurrent bleeding; surgical management remains the only chance of long‐term survival.^6^ However, surgery for secondary AEF is more challenging and still controversial because of the worse hemodynamic status and technical difficulty caused by severe adhesions. In our case, initial aortic stenting for stabilization allowed meticulous adhesiolysis and en bloc resection of AEF. Preventing post‐esophagojejunostomy AEF is an important issue. Recently, minimally invasive surgery and surgical stapling devices have decreased the incidence of anastomotic leakage. However, direct contact between the staple line and thoracic aorta might cause AEF by sustained erosion. Covering the esophagojejunal anastomosis with an omentum flap might be a considerable strategy.^5^

This is a case of post‐esophagojejunostomy AEF who survived more than 2 years. A three‐stage hybrid strategy is safe and feasible to manage fatal secondary AEF, as follows: (1) bridging TEVAR controls bleeding, (2) radical fistula resection eradicates infectious source and fragile structures, and (3) esophageal reconstruction restores gastrointestinal continuity. Most importantly, multidisciplinary approach is the key for success.

## CONFLICT OF INTEREST

The authors declare no conflict of interest.
